# Draft Genome Assemblies of *Enterobacter aerogenes* CDC 6003-71, *Enterobacter cloacae* CDC 442-68, and *Pantoea agglomerans* UA 0804-01

**DOI:** 10.1128/genomeA.01073-14

**Published:** 2014-10-23

**Authors:** T. D. Minogue, H. E. Daligault, K. W. Davenport, K. A. Bishop-Lilly, D. C. Bruce, P. S. Chain, S. R. Coyne, O. Chertkov, T. Freitas, K. G. Frey, J. Jaissle, G. I. Koroleva, J. T. Ladner, G. F. Palacios, C. L. Redden, Y. Xu, S. L. Johnson

**Affiliations:** a Diagnostic Systems Division (DSD), United States Army Medical Research Institute of Infectious Diseases (USAMRIID), Fort Detrick, Maryland, USA; b Los Alamos National Laboratory (LANL), Los Alamos, New Mexico, USA; c Naval Medical Research Center (NMRC), Frederick, Fort Detrick, Maryland, USA; d Henry M. Jackson Foundation, Bethesda, Maryland, USA; e Center for Genome Sciences (CGS), USAMRIID, Fort Detrick, Maryland, USA

## Abstract

The *Enterobacteriaceae* are environmental and enteric microbes. We sequenced the genomes of two *Enterobacter* reference strains, *E. aerogenes* CDC 6003-71 and *E. cloacae* CDC 442-68, as well as one near neighbor used as an exclusionary reference for diagnostics, *Pantoea agglomerans* CDC UA0804-01. The genome sizes range from 4.72 to 5.55 Mbp and have G+C contents from 54.6 to 55.1%.

## GENOME ANNOUNCEMENT

Members of the family *Enterobacteriaceae* are notable opportunistic pathogens, with some species accounting for up to 5% of documented hospital-acquired infections. *Enterobacteriaceae* includes both saprophytic soil and commensal enteric microbes. Many have become troublesome nosocomial pathogens, with high resistance to β-lactam antimicrobials (1). In this study, we sequenced two reference strains of *Enterobacter* (*E. aerogenes* ATCC 29904 and *E. cloacae* ATCC 13047), as well as a near neighbor and plant commensal, *Pantoea agglomerans* UA 0804-01 (1, 2).

High-quality genomic DNA was extracted from purified isolates of each strain using Qiagen Genomic-tip 500 at the United States Army Medical Research Institute of Infectious Diseases-Diagnostic Systems Division (USAMRIID-DSD). Specifically, 100-ml bacterial cultures were grown to stationary phase and nucleic acid extracted as per the manufacturer’s recommendations. These draft genomes included a combination of Illumina (3) and 454 technologies (4). For each genome, we constructed and sequenced a 100-bp Illumina library (300- to 733-fold genome coverage) and a long-insert paired-end 454 library (6,936- to 7,528-bp inserts at 14- to 35-fold genome coverage). The 454 paired-end data were assembled in Newbler (5), and those consensus sequences were computationally shredded into 2-kbp overlapping fake reads (shreds). The Illumina sequencing data were assembled with Velvet (6), and the consensus sequence was computationally shredded into 1.5-kbp overlapping shreds. All data were additionally assembled with AllPaths (5), and the consensus sequences were computationally shredded into 5-kbp overlapping shreds. We integrated the 454 Newbler consensus shreds, the Illumina Velvet consensus shreds, AllPaths consensus shreds, and the 454 paired-end library read pairs using the Phrap parallel version (High Performance Software, LLC). Possible misassemblies were corrected using in-house scripts and/or manual editing in Consed (7–9).

Automatic annotation for each genome utilized an Ergatis-based (10) workflow at the Los Alamos National Laboratory (LANL), with minor manual curation. Table 1 lists the basic genome statistics and annotation counts. The final annotated assemblies are released in NCBI, and the raw data can be provided upon request, with a preliminary review of the annotated genomes and located β-lactam resistance genes in each.

**TABLE 1:** Basic assembly and annotation statistics for the three *Enterobacteriaceae* genomes

Species	Strain	Alt ID^*a*^	ATCC	Accession no.	Length (bp)	No. of:					G+C content (%)
						CDSs	Scaffolds	Contigs	rRNAs	tRNAs	
*Enterobacter aerogenes*	CDC 6003-71	EAX	ATCC 29904	JOUC00000000	5,084,129	4,692	1	9	19	84	55.1
*Enterobacter cloacae*	CDC 442-68	EBB	ATCC 13047	JPPR00000000	5,551,574	5,393	4	43	14	85	54.6
*Pantoea agglomerans*	CDC UA0804-01	EAY	NA^*b*^	JOUD00000000	4,720,650	4,494	2	9	17	83	54.8

a Alt ID, alternative identification.

b NA, not applicable.

### Nucleotide sequence accession numbers.

The NCBI accession numbers are JOUC00000000 for *E. aerogenes* CDC 6003-71, JPPR00000000 for *E. cloacae* CDC 442-68, and JOUD00000000 for *P. agglomerans*.
